# Opinions of South African physiotherapists on gross anatomy education for physiotherapy students

**DOI:** 10.4102/sajp.v75i1.1318

**Published:** 2019-07-30

**Authors:** Dorothy Shead, Ronel Roos, Benita Olivier, Amadi O. Ihunwo

**Affiliations:** 1 Department of Physiotherapy, University of the Witwatersrand, Johannesburg, South Africa; 2 School of Anatomical Sciences, University of the Witwatersrand, Johannesburg, South Africa

**Keywords:** gross anatomy, curriculumnatomy, pedagogynatomy, physiotherapynatomy, physiotherapistsnatomy, physiotherapy students

## Abstract

**Background:**

Physiotherapists know the depth of gross anatomical knowledge required for safe and effective clinical practice. They can offer insightful opinions on inclusions for and teaching of an anatomy curriculum for physiotherapy students.

**Objectives:**

The aim of this study was to gather opinions of physiotherapists as to what they perceive as necessary anatomy curricular content for undergraduate physiotherapy students and identify pedagogy that should be used.

**Method:**

A qualitative methodology using a grounded theory approach incorporating semi-structured interviews was utilised in this study. Theoretical sampling was used to identify representative South African physiotherapists. An inductive process, using continuous manual analysis of data by two independent coders, was undertaken. Data were collapsed until themes were identified. Triangulation and other strategies for trustworthiness of data were instituted.

**Results:**

Theoretical saturation was reached after five focus groups (*n* = 32). Demographical information indicated physiotherapists of all age groups and both genders working in diversified clinical areas. Seven themes were identified and incorporated information from ‘structure’, ‘content’ and ‘pedagogy’ for anatomy programmes to the psychological impact of course aspects on a student’s psyche. Vertical integration of anatomy into later preclinical years, incorporation of physiotherapists to teach anatomy, a ‘physiotherapist personality’ and ‘anatomy know how’ for clinical practice were included.

**Conclusion:**

Opinions of physiotherapists are important in identifying curricular and teaching considerations that can be incorporated into an anatomy programme designed for physiotherapy students.

**Clinical implications:**

Targeted anatomy education for physiotherapy students can aid learning and retention of anatomical knowledge necessary for effective and safe clinical practice.

## Introduction

Physiotherapy (PT) is a clinical profession aimed at maximising clients’ ability to move and function when they have been affected by ageing, physical injury, diseases and disorders (World Confederation of Physical Therapy [WCPT] 2016). Physiotherapists in South Africa are first-line autonomous practitioners (WCPT 2011) and can diagnose and treat a patient’s condition within their scope of practice. South African PT students learn anatomy as a basis for clinical practice (Shead et al. 2018). Therefore, a gross anatomy curriculum should be clinically relevant to PT practice (Moxham et al. 2014). Aspects to consider when developing anatomy teaching are the role of PT professionals, course content and type of pedagogy (Kleibard 1989).

The published scoping review protocol by Shead et al. (2016) led to the realisation that there was a paucity of information in relation to how anatomy was being taught to PT students globally. The resultant scoping review by the same authors identified that there was a wide variety of curricular approaches and pedagogies being utilised. It also reported on the use of clinical vertical and horizontal integration scenarios in the PT anatomy curriculum illustrating the link between anatomical knowledge and clinical application. The number of studies sourced (*n* = 52) illustrated that there was a paucity of information on the topic published over four decades (1980 and 2018). The facts gleaned from that study provided impetus for the conduction of the focus group (FG) interviews in this study in order to gain additional information on the state of gross anatomy education in South Africa.

Concurrently, a survey by Shead et al. (2018) investigating how gross anatomy is taught to PT students in South Africa found that there was no unified anatomy curriculum across the country. Furthermore, the Health Professions Council of South Africa (HPCSA) has not defined an anatomy curriculum for PT students. The survey highlighted some areas of concern pertaining to limited or non-existent access to computer technology and large student groups leading to poor staff to student ratios in half of the responding faculties. A wide range of 100–308 anatomy direct contact hours was identified between the six responding faculties together with varied pedagogies and learning techniques. Hence, disparity exists across the country.

However, there is no literature to verify whether PT standards are impacted by the situation. Focus group interviews with physiotherapists working in service delivery can further delve into these differences and give insight into which curricula and pedagogies are best suited to achieve optimal gross anatomy education for PT students in South Africa and enable suggestions for a unified curriculum. The purpose of our study, therefore, was to establish what physiotherapists in different service delivery sectors in the Gauteng province of South Africa perceived as necessary content for a gross anatomy curriculum and pedagogy for South African PT students.

## Method

A qualitative study with FG interviews was undertaken using a grounded theory approach and the opportunity to participate in this study was open to all physiotherapists employed in service delivery sectors in the Gauteng province of South Africa.

Physiotherapists in public or private practice and PT lecturers teaching PT students at the University of the Witwatersrand (WITS) were eligible for inclusion. No exclusion criteria were applied for eligible participants. Individuals were invited to participate in the FGs if they had the qualifications to practise as physiotherapists. Demographic information collected prior to all the FGs indicated that this was the case. The sampling strategy did not aim to get physiotherapist representation from all of the relevant eight health science faculties in South Africa but the resultant sample was representative.

Sample size was dependent on the number of eligible participants to be sampled in order to reach theoretical saturation (Glaser & Strauss 1967). Theoretical sampling was used to gather participants (Cresswell & Poth 2018). In total, five FGs were conducted using a total of 32 participants from various PT service delivery sectors.

Patton and Cochran (2002) state that an ideal FG should contain 6–10 persons. Two FGs were conducted with six, one with seven and one with eight participants. The first FG (*n* = 5) constituted a pilot study and was within guidelines advocated by Morgan (1998). The pilot group collected useful information and formed a good foundation for the continuation of the grounded theory methodology. Hence, the collected data were included in the analysis of the results.

A SASP bulk email was sent to all registered physiotherapists in the Gauteng province of South Africa followed by engagement with private practices, public hospital PT departments and the WITS PT department to invite participation. Participants within each sector had an interest in specific areas of PT practice. This factor allowed a skewed perspective from theoretical sampling to be avoided.

### Data collection

Data collection was driven by the principles of grounded theory and conducted between September 2016 and May 2017. Grounded theory is defined as an emergent method and as such it is open-ended and has no determinate format (Charmaz 2008). In grounded theory, data collection and analysis are carried out simultaneously in order to allow a continually changing response to theoretical sampling (Corbin & Strauss 1990). In this study, data were collected using open-ended questions that were formulated and adapted according to the direction that a FG discussion was taking and were further adapted for subsequent FG discussions because of on-going analysis of data using an inductive methodology (Cresswell & Poth 2018). The FG interviews were continued with the intention of achieving theoretical saturation if/when no more information was being offered on that particular topic (Glaser & Strauss 1967). The FG process was continued until the collected data were saturated after five FGs. Hence, the study adhered to grounded theory principles.

The first author (facilitator) conducted all five FGs. An opening question of ‘Please tell me about the way that you were taught gross anatomy when you were an undergraduate physiotherapy student?’ was used to initiate FG discussions. Thereafter, questions were modified according to the flow of the conversation in the FGs. Field notes were taken and a reflective process of ‘personal memo writing’ was undertaken (Lempert 2007:245). Discussions were held in quiet conveniently located venues. Circular seating ensured easy interaction between the facilitator and participants.

### Data analysis

Descriptive data analysis was done using IBM SPSS 24 statistical package, version 24.0 (IBM Corp., Armonk, NY) to provide information on the demographic profile of study participants (Table 1).

**TABLE 1:** Demographic characteristics of study participants (*N* = 32).

Variable	Public (*n* = 14)		Private (*n* = 11)		Academic (*n* = 7)	
	Variable	%	Variable	%	Variable	%
**Age**						
Range in years	22–31	-	25–48	-	25–47	-
Median years	23.5	-	30.0	-	33.0	-
Interquartile range (IQR)	2.0	-	13.3	-	7.3	-
Mean years	24.2	-	34.6	-	33.9	-
Standard deviation (s.d.)	± 2.4	-	± 8.0	-	± 7.8	-
**Physiotherapy experience**						
Range in years	1.0–2.5	-	2.5–25	-	3.0–25	-
Median years	1.0	-	8.0	-	8.0	-
Interquartile range (IQR)	1.0	-	10.0	-	8.8	-
Mean years	1.4	-	12.7	-	11.1	-
Standard deviation (s.d.)	± 0.6	-	± 7.3	-	± 8.1	-
**Gender *n***						
Male	2	14.3	2	18.2	2	28.6
Female	12	85.7	9	81.8	5	71.4
**Physiotherapy qualification *n***						
Bachelor’s degree	14	100.0	6	54.5	2	28.6
Master’s degree	-	-	5	45.5	3	42.9
Doctorate	-	-	-	-	2	28.6
**Clinical work area *n***						
Neuromuscular-skeletal	5	36.0	7	64.0	3	43.0
Orthopaedics	3	21.0	7	64.0	-	-
Paediatrics	5	36.0	5	45.0	2	29.0
Adult neurology	5	36.0	1	9.0	2	29.0
Cardiopulmonary	1	7.0	4	36.0	-	-
Community Health	-	-	-	-	1	14.0

IQR, interquartile range; s.d., standard deviation.

An inductive method was utilised to analyse qualitative data from the FGs (Cresswell & Poth 2018). The facilitator undertook verbatim transcription in Microsoft Word timeously after each FG. Transcribed data were analysed and coded using manual qualitative analysis undertaken by the facilitator and another independent researcher, designated the first and second coder, respectively. Two phases were allotted to coding: initial line-by-line coding followed by focussed coding (Charmaz 2008). Finally, selective coding was applied where the identified categories were linked to a central phenomenon in a systematic fashion.

Validation of relationships was achieved and fine-tuning of categories occurred with further development and refinement (Corbin & Strauss 1990). Codes were collapsed into subcategories and then aggregated into categories as a means of identifying final themes (Smith 2000). As data were collected coded comparison of themes occurred to identify if they conflicted or agreed with others. Initially, the two coders conducted this process independently. Subsequently, a meeting between the two coders occurred where comparison and discussion of findings resulted in the finalisation of themes. Data that did not follow the main themes were not discarded (Portney & Watkins 2009). These data were not reported on in detail and were tabulated and stored safely. Once no more new concepts or relationships emerged and the data were repetitious theoretical saturation was reached. Inter-coder reliability strengthened the process because of ‘reproducibility across coders’ (Krippendorff 2004:Chap 11). Inherent in this procedure was the principle of ‘bracketing’ as described by Rolls and Relf (2006) where the coders purposely ignored their own feelings on what was being revealed in the data analysis. Therefore, they proceeded with an open mind without the occurrence of bias.

### Trustworthiness

Analyst triangulation was achieved by the use of two independent coders. Using triangulation of sources the PT service delivery FGs provided different data sources within the same method. This was achieved by exclusive collection of data in each service delivery sector FG separately before later integration of data. Hence, this method provided more than one source of data within the same collection of data process (Krefting 1991). The collection of data using a recording device and field notes assured methods triangulation. The grounded theory approach ensured that an auditable record of data collection was maintained and thus provided confirmed ability of strategy (Guba 1981). The trail of research methodology was laid out for each part of the FG process in detail to ensure dependability (Guba 1981). Furthermore, persons in a FG should be typical and representative of the population of which they are members (Field & Morse 1985). All participants were qualified physiotherapists and over the five FGs all eight of the universities offering PT gross anatomy programmes in South Africa were represented.

### Ethical considerations

All FG participants signed an informed consent form allowing for the use of a tape recording device. Ethical clearance for the study was obtained from the Human Research Ethics Committee (Non-Medical) of the University of the Witwatersrand, Johannesburg, South Africa (H15/04/12).

## Results

### Demographic information of focus group participants

In terms of service delivery sectors, FGs 1 and 3 were from the private sector, FGs 2 and 4 from the public sector and FG 5 was from the academic sector. Demographic data from the five FGs were pooled and analysed in relation to PT service delivery participants. Table 1 shows that the public sector FGs had the youngest and least experienced participants as 50% of these physiotherapists were doing their community service year. Although clinically inexperienced, younger physiotherapists added value to the study with their insights into how recent gross anatomy education prepares a physiotherapist for clinical practice. Higher degrees were associated with sectors that had increased years of PT work experience. The majority of physiotherapists in each sector were female. Table 1 also records the diverse clinical areas in which FG participants work and percentage participation indicated that some worked in more than one clinical field.

Seven themes were identified during the data analysis. Table 2 illustrates the categories and subcategories related to each theme:

- The ‘Bare bones’ of anatomy
- Anatomy: ‘A touching experience’
- ‘Staff matters’ in anatomy
- ‘Student embodiment’ in anatomy education
- Gross anatomy classes
- ‘Time is of the essence’
- ‘Anatomy know how’ for physiotherapy practice

**TABLE 2:** Identified themes, categories and subcategories.

Theme	Category/categories	Subcategories
1. The ‘Bare Bones’ of Anatomy	Anatomy teaching methodology	Teaching activities and materials When Content Structure Course objective Duration and frequency
2. Anatomy ‘A Touching Experience’	Student readiness Student assessment Anatomy teaching methodology	Impact of dissection on student psyche Emotional response to assessment Teaching activities and materials. Emotional response to course content and timing
3. ‘Staff Matters’	Matters relating to staff teaching	Motivation of staff Quality and quantity of staff Relevance of professional affiliation of staff Staff continuity Staff language and communication
4. ‘Student Embodiment’ in Anatomy Education	Inter-professional education Student assessment Student readiness Ethical considerations	Between student disciplines Order of alignment of content with assessment Timing of assessment Positive assessment Negative assessment Maturity and responsibility Student’s experience of course content, motivation assessment and perception Learning techniques Ethics Cultural and religious beliefs
5. Gross Anatomy Classes	Characteristics of classes	Size of classes Size of dissection/prosection group and interaction between group members Chronological changes in size of classes
6. ‘Time is of the Essence’	Time allocated to cover the curriculum	Time available for course versus pressure on students to learn
7. ‘Anatomy Know How’ for Physiotherapy Practice	Clinical implications Physiotherapist’s psyche Integration	Clinical relevance and anatomy The ‘physiotherapist personality’ Anatomy integration into physiotherapy course

### The ‘Bare bones’ of anatomy

*Dissection* was the most frequently identified code in this theme (*n* = 24, 41%). With regard to teaching activities and materials, the three-dimensional (3D) value and whole body perspective of dissection was appreciated. Some participants observed prosected cadavers. Observing a correctly prepared prosection was identified as a way to identify structures cut away during student dissection. Materials used to teach students included plastinated specimens, textbooks, atlases, videos, blackboards and human bones. Visiting an anatomy museum was suggested as useful for pretest revision. Computer technology was identified as an adjunct pedagogy. Online learning was acknowledged and the use of ‘Apps’ was recommended. Lectures were considered tedious with too much information.

In the sub-category content, *muscle* and *spinal cord* were the most frequently identified codes (*n* = 19, 23%).

The brain and lungs were also highly rated. Inclusion of detailed anatomy of the abdomen was not felt necessary.

The setting of goals was identified as a way to achieve course objectives. There was a wide range of variation in time spent in and frequency of dissection and prosection, use of computers, tutorials or lectures.

The most common distribution of a course was into modules associated with a regional approach or quarters associated with a systemic approach. Participants either had first year anatomy, second year anatomy or anatomy over 2 years.

Sub-category teaching activities and materials:

‘(Referring to dissection) I think it helped me… visualise all the layers… just to say that soleus is behind gastrocs’. (FG2, Quote 155)

‘… It also gives you an idea of how everything fits in together ….’ (FG1, Quote 53)

‘We used to do our own dissections but… in the front of the hall… we had a cadaver that was done properly so like not us tearing and ripping and cutting and slipping and slicing… You could go and look at… so that was slightly more useful… when I felt I don’t know in dissection.’ (FG4, Quote 41)

‘(Referring to the museum) …We call it … the “biltong book” … the lecturer would go … a week before the test … move things around so… it’s all the upper limb things… displayed and marked so that helped a lot ….’(FG4, Quote 36)

‘Because you’re not treating your computer … it changes it completely … even… your cadaver, it doesn’t look exactly the same and the position isn’t exactly the same … But it’s a good add on though.’ (FG1, Quote 262)

‘Instead of just get another slide with the shoulder on it and the muscles pointed out and innervation… there’s tons of apps that you could show students.’ (FG1, Quote 253)

‘Online learning?’ (FG3, Quote, 244).

‘We did have it’. (FG3, Quote 247)

‘Very long, tedious with lots of information lectures…that I used to fall asleep in ….’ (FG5, Quote 20)

Sub-category content: Table 3 illustrates the acceptable and unacceptable content for a PT gross anatomy curriculum with relevant quotations.

**TABLE 3:** Content of a physiotherapy gross anatomy curriculum.

Preferred content	Optional content	Focus group quotation
Musculoskeletal	-	‘We did a lot on musculoskeletal stuff’ (FG1, Quote 22)
Spinal cord	-	‘…I think definitely more spinal cord things …’ (FG3, Quote 154)
Nerves	-	‘If you have followed the sciatic nerve from its origin to insertion’ (FG5, Quote 60) ‘… central nervous system and nerves and peripheral nerves…’ (FG1, Quote 44)
Brain	-	‘But I think they did too little with the brain …everything is connected to the brain. I think it should be like the first thing that we learn …’ (FG4, Quote 110)
Upper limb and lower limb	-	‘They spent most of the time for us on upper limb and lower limbs… So that was nice’ (FG5, Quote 135)
Brachial plexus	-	‘ I can still understand the brachial plexus and why that would be important to me…’ (FG3, Quote 41)
Lungs	-	‘The lungs are really important for us like to learn…’ (FG2, Quote 146)
Back	-	‘… I would have loved to have dissected more with the back’ (FG3, Quote 181)
Fascia	-	(Referring to fascia) ‘… If I could just actually look at how that looks on that muscle as I’m cutting it away …see how it goes through the muscle the way we describe it now I’d appreciate that SO MUCH MORE …’ (FG3, Quote 191)
Bones/osteology	-	‘… the first quarter was the bones’ (FG2, Quote 14)
Medical terminology	-	‘I think that we should be learning medical terminology’ (FG1, Quote 46)
-	Intestines	‘… I think… learning about all the intestines … I think we went into way too much detail’ (FG2, Quote 146)
-	Mandible	‘Why are we focussing on a mandible?’ (FG2, Quote 41)
-	Face and Internal organs	‘We didn’t dissect face and we didn’t dissect internal organs’ (FG 3, Quote 63)

Sub-category course objective:

‘…We were given … goals to meet each week where we had to dissect…for example…the shoulder by end of week two, elbow by week three….’ (FG3, Quote 13)

### Anatomy: ‘A touching experience’

The touching experience refers to not only the physical touching as in surface anatomy but also the emotional touching of the student psyche. Although the latter is not often considered in curricular matters, the impact of emotional responses to anatomy course content and assessment can influence student learning. Furthermore, some students feel pressure when first encountering a cadaver. Desensitisation rituals can help ease the experience. The amount of anatomy to be learnt in a limited timeframe can generate emotional responses from students.

Impact of dissection on student psyche:

‘When we started with anatomy… There was a cadaver on a table with a cloth over and the whole lecture was just to get everyone desensitized and comfortable and it was very respectable… I think that definitely helped….’ (FG1, Quote 62)

Emotional response to course content and timing:

‘I would cry about that thing and …I NEVER CRY!…the load…it’s so much and they cram it all into… one year.’ (FG 4, Quote 187)

Teaching activities and materials (surface anatomy):

‘… So we were in groups and we had to draw on each other… different muscles and they would correct us and I found that really useful.’ (FG2, Quote 166)

Emotional response to assessment:

‘… Spots … it was helpful but … that little time that you are given …It’s very difficult to spot a muscle … nerves… arteries… veins and… that time …it’s so little …five seconds or something like that….’ (FG4, Quote 105)

### ‘Staff matters’

Some lecturers were described as *passionate* or *gurus* and others as only doing the job because it was required of them. Lack of staff continuity was found to be disruptive and made learning difficult. Poor staff to student ratios and inadequately qualified staff were highlighted. A participant noted that if a lecturer was a ‘foreigner’ and ‘very heavily accented’ students had a problem understanding what was taught during anatomy. Relevance of the professional affiliation of staff was linked to making anatomy more clinically relevant.

Quality and quantity of staff:

‘I think we had teaching assistants… but they didn’t really help much. I don’t think they had good training….’ (FG1, Quote 97)

Relevance of professional affiliation of staff:

‘Our table doctor was a physio so she related a lot of what we were seeing and doing to patients we would see in the future so that made it quite relevant to us….’ (FG5, Quote 33)

### ‘Student embodiment’ in anatomy education

It was seen that in the majority of cases PT and occupational therapy (OT) students learnt together.

Participants felt that anatomy teaching in the inter-professional scenario should still address future PT clinical relevance. Learning by doing and peer teaching were suggested as successful learning techniques. A participant described rote learning as a poor strategy for anatomical knowledge retention. With regard to assessment, if participants had been examined on something that they had not yet learned in class a negative assessment experience was reported. However, participants who were tested on current work reported a positive assessment experience. Sectional tests in either the written, practical or multiple choice question formats were spread throughout course structure and final exams were held at the end of the course in every instance reported. Poor knowledge of ethical matters was raised especially in relation to the photographing of cadavers. However, some participants described their anatomy experiences with a strict dress code and defined ethical boundaries. It was apparent that students in anatomy programmes came from diverse religious and cultural backgrounds. Such factors had to be considered in surface/living anatomy sessions. It was mentioned that mainly male class members would undress for surface anatomy. Students who for religious or modesty reasons needed privacy could participate behind a screen or are excused from the class.

Between student disciplines:

‘…With anatomy it was never shown as to how it applies to us because we did it with OT, pharmacy, dentistry. It was never how does this help you as a physio.’ (FG4, Quote 46)

Learning techniques:

‘…I think anatomy is very intimidating… you get a huge text -book and then you think Oh my word! How am I going to learn this? … then you parrot learn so you can pass. You’re actually learning …ABSOLUTELY NOTHING…’cos then next week if someone asks…you won’t know….’ (FG4, Quote 60)

Ethics:

‘I think it would make much more sense if they would just brush through the ethical biography. Explain that you’re not allowed to take pictures….’ (FG4, Quote 196)

### Gross anatomy classes

A progressive chronological increase in class size was noted. The increase in size of dissection/prosection groups corresponded with the increase in class sizes and difficulty with dissection/prosection participation in larger groups was noted. Class sizes were described as too big in the majority of cases. Large class size was associated with difficulty in hearing an instructor during a tutorial. Group dynamics differed and in some cases one person took charge whereas others just observed or chatted. However, one participant reported an ordered rotation of duties during dissection.

Chronological changes in size of classes:

‘If we’re on the actual class size. I mean when I did physio I was one of 29. Now the physio class is like 60–70. So I mean the classes…the groups are going to be a lot bigger.’ (FG3, Quote 315)

### ‘Time is of the essence’

The codes *all bombarded into second year* and *too much content for one year* give a perspective on the pressure that students feel because of the amount of anatomy to be learnt in a finite period of time. A participant expressed the pressure to learn as:

‘… and you’re just trying to squeeze as much as you can into such a short period ….’ (FG4, Quote 60)

### ‘Anatomy know how’ for physiotherapy practice

Mention was made of the many PT specialities and the fact that the anatomy course has to cater for every eventuality. The responsibility of first-line practitioner status of a South African physiotherapist was raised in connection with the clinical need to have adequate anatomical knowledge. A participant gave insight into the fortuitous timing of biomechanical PT practical sessions with anatomy course content. A physiotherapist personality type was described which identified anatomy lectures as not being optimal pedagogy for physiotherapists who are practically orientated people. Participants alluded to the necessity of a good knowledge of surface anatomy in relation to clinical scenarios.

Anatomy integration into PT course:

‘… We overlapped our biomechanics… When you presented your muscle you’d go as fine as possible into the anatomy and then you’d do the surface anatomy on your model and then you’d do your testing.’ (FG1, Quote 49)

Clinical relevance and anatomy:

‘… We start going to clinicals from second year …I can see patients and see injuries although we’re just doing …subjective assessments but you’re hearing things that he brings up…. Only then did I start to appreciate it.’ (FG3, Quote 45)

‘I was working with a quadriplegic patient… his first rib was… really sensitive. He was complaining of… cervical pain… and he said “What are you doing?” “and I said …I’m mobilising your first rib” “My first rib? It’s not there!” “I rushed off to get my anatomy book and show him where it is…” “You need to know where to find the first rib …it’s a very good thing I did surface anatomy”.’ (FG1, Quote 219)

‘In the end the patients we’re working on are living. We can’t dissect them to see what’s going on inside. We need to be able to identify the landmarks.’ (FG3, Quote 259)

## Discussion

### Curriculum

In medical education, less anatomy instructional time has been noted (Drake, McBride & Pawlina 2014) and yet the basic anatomy content has not decreased (Pawlina & Drake 2017). No published evidence confirms that gross anatomy time has decreased for PT students but this study attests to this point. Participants related emotional distress in relation to the anatomy load being crammed into a year or talked about trying to squeeze as much information into ‘such a short period’. Extra topics added to the overall PT course curriculum because of the advent of PT practice specialisation, may have impacted time allocated to normal anatomy. The delivery of the anatomy curriculum over 2 years that some participants experienced was perceived as a less stressful way to learn anatomy. Hence, this may be one aspect of the disparity between faculties that could be resolved in favour of a 2-year anatomy programme. Other factors identified as influencing availability of instructional time are financial constraints and fewer suitably qualified anatomy teachers (Gabard, Lowe & Chang 2012).

There has been a chronological increase in anatomy class sizes (Zhang 2017). The difference in class sizes reported by Reimer, Laurenzo and Tages (2013) – between 31 and 50 – and Shead et al. (2018) where mean class size was 60.5 (±12.6) further illustrates the point. This has impacted dissection/prosection group sizes as reported in this study. The number of students per cadaver is increasing chronologically: 1:4 (Cantor 2010:180), 1:5 in Kenya (Ongeti 2012) and 1:8–9 in Australia (Dissabandara et al. 2015) and 1:8 (±3.5) in South Africa (Shead et al. 2018). These authors also found a distribution of between 4–6 and 10–12 students to cadaver compared with that reported by Reimer, Laurenzano & Tages (2013) as 4–6 in 87.5% of faculties. Increase in class sizes will have a serious impact on curricular choices in the future.

Zhang (2017) noted the increase in class sizes means each faculty member will have to cater to the needs of more students. Our study found that there was dissatisfaction with staff to student ratios. Unsatisfactory staff knowledge, lack of staff continuity and difficulty understanding staff with foreign accents was also raised. Some authors maintain that PT practitioners should teach the gross anatomy curriculum for PT students in order to safeguard the clinical element (Reimer et al. 2013). Focus group members felt that suitably qualified PT staff would be preferable gross anatomy instructors as they offer interesting clinical insight during anatomy sessions.

There is a paucity of evidence relating to preferred anatomy curricular content for PT students and much of it is out-dated. Important content was identified as the brachial plexus (98.9%), spinal cord (94.7%), lower limb, thorax and brain (69.2%) and least important content related to the abdomen and aspects of the pelvis (Mattingly & Barnes 1994). Students in Canada and the USA spent 46% and 48%, respectively, of their gross anatomy course studying the upper and lower limbs (Armstrong & Rosser 1996). Latman and Lanier (2001) reported that PT clinicians favoured the inclusion of the nervous and muscular systems and rated coverage of the lower limb and back equally. Youdas, Krause and Hellyer (2015) stated that the Mayo Clinic PT anatomy programme emphasised the musculoskeletal aspects of the subject and included the lungs. Focus group results mimicked the aforementioned rating of content by the above authors. Like Youdas et al. (2015) and Mattingly and Barnes (1994) FG participants stated that detailed coverage of the intestines was unnecessary. The rating of the importance of content inclusion assists in the allocation of time to that region or system in the anatomy curriculum.

The regional and the systemic approaches or a combination of both are used to teach anatomy. Dissection is usually associated with a regional anatomical approach (Pais & Moxham 2013). It was therefore not surprising that most FG participants had used a regional approach as they did dissection. However, dissection is a time-consuming activity and time allocation for anatomy is decreasing. Pather (2015) stated that prosections are time-efficient, flexible, allow for easy recognition of anatomical structures and associated relations and can be viewed in context with fewer cadavers needed to be used for observation by students. The availability of cadavers (Gangata et al. 2010) and costs of preparing and storing cadavers may influence pedagogy choice in some faculties.

The context in which a student learns anatomy impacts on how well they learn the subject (Smith, Martinez-Álvarez & McHanwell 2014). The FG consensus was that learning anatomy ‘parrot fashion’ to pass exams was a bad strategy and did not help with recall. Peer teaching was described as beneficial as was visiting the anatomy museum described graphically in the ‘Biltong Book’ experience. The topic of inter-professional education is covered in global literature in relation to its value to collaboration and teamwork (Fernandes et al. 2015; Meyer et al. 2017) and understanding of other disciplines’ professional role (Sytsma et al. 2015).

In this study, PT students mainly learnt with OT students and Shead et al. (2018) found the same situation. The concern raised in the FGs regarding inter-professional education was that the anatomy was not specifically geared towards clinical preparation for PT students.

Participants described a negative reaction to time allocated for spot tests and the consequential pressure felt by students. Our study discovered student assessment was formative using progressive tests and/or summative assessment at the end of it as suggested by Mitchell and Mitchell (1998). These authors also suggested that if a student attained a 60% average mark for the year exemption should be considered. FG participants were positively aware of and disposed to this concept. The appropriate timing of examination with the course content elicited a positive assessment experience for participants. However, with poor timing negative assessment experiences were felt. Hence, the type of examinations, timing of examinations and content of examinations have to be carefully factored into the curriculum to produce optimum outcomes.

The FG discussions identified the effect of dissection/prosection on the student psyche. This phenomenon has to be considered when considering how to structure desensitisation measures into a curriculum. Jones, Lachman and Pawlina (2014) described how donors were honoured in memorial ceremonies in the United States.

Participants remembered solemn dedication ceremonies for cadavers. The standardisation of gross anatomy ethical guidelines for students has been proposed (Kahn & Gardin 2016). Participants suggested that ethical guidelines should be clearly defined at the start of the programme to avoid students transgressing.

### Pedagogy

It has been said that human dissection is incomparable for the type of anatomy education that acknowledges the importance of ‘the feel of human flesh’ (Azer & Eizenberg 2007). Reimer et al.’s survey (2013) of PT gross anatomy programmes in the United States recorded that 92.6% of the respondents used dissection in their faculty. Participants recalled the 3D appreciation of depth of and anatomical relationships between structures afforded by dissection. Harper, Steinbeck and Aron (2019) reported that deep fascial manipulation might be an effective PT treatment for low back pain. Adstrum (2017) concluded that traditionally dissection ignored the 3D, dynamic fascia component of the body. Participants described the traditional way of removing fascia during dissection and suggested that attention should be given to more observation of facia *in situ* and realisation of its clinical importance.

Some FG participants learnt anatomy by observing prosected cadavers. The availability of a prosected cadaver also helped students who did not dissect well to observe structures removed accidentally. However, Stabile (2015) reported that the systemic approach might be problematic for precise identification of structures and the interrelationship between them.

Using a body painting exercise, Nanjundaiah and Chowdapurkar (2012) taught surface anatomy of the upper limb to PT students. The experience proved effective. Although surface anatomy aids learning, the impact of religious and cultural factors can influence its implementation. The literature records that most female Muslim students cite religion as the reason for not wanting to take part in peer surface anatomy sessions (Das, Townsend & Hasan 1998). A similar situation was reported in the FG groups where students participate in surface anatomy sessions within a comfort zone dictated by religious choices. However, Azer (2013) found surface anatomy methods such as using cadaver palpation, full body radiographs, body painting and virtual tools can be used to overcome barriers to participation. The relevance of surface anatomy knowledge as regards clinical competency was illustrated by FG participants’ comments, so it is an important pedagogical inclusion.

The duration and frequency of anatomy lectures, tutorials, dissections and prosections varied widely across the different programmes. A participant recalled long, tedious lectures. The identified ‘physiotherapist personality’ was not well predisposed to lectures. Hence, blended learning with fewer lectures as instituted by Green and Whitburn (2016) in Australia might offer a better alternative. However, Shead et al. (2018) found that in South Africa 50% of the health science faculties that responded to their survey could not supply computers for student use. Therefore, the use of blended learning in those faculties is not feasible.

Smith et al. (2018) found the use of 3D printed models in a small group scenario, by students at university and at home, was beneficial to learning on a particular topic. While this mode of delivery of anatomy knowledge has many benefits, the ethical issues relating to the use of cadaveric remains as a source for reproduction of body parts still need to be addressed (Jones 2018). There is no available literature as to the use of 3D printing in anatomy education for PT students.

Krause, Youdas and Hollman (2011) put forward that integrating anatomy during the entire PT course helps to reinforce previously learnt anatomical material. They conducted a vertical integration study where students tested upper and lower limb joints on a cadaver. Studies that incorporated horizontal integration of clinical experiences in anatomy included the performance of the Lachman test on lightly embalmed cadavers (Janssen et al. 2014), an integration of the presentation of a clinical case with demonstrations on prosected cadavers of related respiratory anatomy (Parmar & Rathinam 2011) and a study with the horizontal integration of neuro-anatomy with the presentation of four neurological cases (Prithishkumar & Holla 2012).

Students enjoyed these experiences, improved retention of anatomy knowledge and gained clinical confidence.

## Conclusion

The findings of this study confirm that a modern anatomy curriculum for PT students has to be adapted to cope with time constraints that impact pedagogy selection and learning strategies. In this regard, dissection is still preferred but decreased time might lead to prosection or computers being used more frequently.

However, FG participants did not favour the use of computers as a main pedagogy. A chronological increase in the size of classes and tutorial and dissection/prosection groups was noted with a resultant increase in student to cadaver ratios. Lack of recruitment and retention of staff because of financial constraints will result in increasing low staff to student ratios as noted by FG participants and inefficient anatomy delivery. There was also a preference for staff to be suitably trained physiotherapists to enhance the clinical aspects of the programme. Students’ emotional ability to master the anatomy load must be considered when deciding the nature and amount of anatomical content to be delivered in the available time. Desensitisation should occur to help students cope with the introduction to cadaveric remains. Blended learning as a unified curricular concept was identified as not feasible because of a lack of computers in South African faculties. The limitations of this study are in the fact that the clinical viewpoint of the younger physiotherapists is limited and the academic FG viewpoint was only garnered from one university. Integration of anatomy into a PT curriculum and development of a unified anatomy curriculum remains a challenge for South Africa. It is recommended that future research should focus on gaining input from experienced physiotherapists exclusively and collecting data from other academic institutions on this evolving topic.
